# Elegant Pharmacy

**Published:** 1882-05

**Authors:** 


### Editorial.

### ELEGANT PHARMACY.

In these days of enterprise and ingenious development, it does not come amiss to halt occasionally in the impetuous haste which marks the progress of the times, and cautiously surveying our surroundings, to reckon our latitude and longitude as measured by established and familiar landmarks.

It must be acknowledged that the principal aim of the physician in the pursuit of his profession is the cure of disease. Hence the means within his reach—the implements of his art, if you choose—should be, as far as possible, trustworthy and absolutely free from flaw, imperfection or impurity. Any unnecessary or any avoidable risks which the practitioner incurs by the use of remedies of doubtful or uncertain quality is unwarrantable, and the custom of taking extraordinary risks of this character is unworthy of a conscientious and intelligent man. We candidly admit the advantages which accrue from the skillful compounding of drugs, but this skill should be possessed and exercised by the prescriber, and this care should be exhibited by himself or his trusted agents, so that all elements of uncertainty which it is possible to avoid may be eliminated; and it should not be forgotten in striving to overcome the unpalatable qualities of medicines, that a disagreeable flavor, while it is sometimes a serious hindrance to the satisfactory administration of certain valuable remedies, is, nevertheless, as a rule, only incidental, and of secondary importance. The principal object is to secure the remedial effect, and if the virtues of the article are impaired or destroyed by efforts to disguise its taste or to conceal its characteristics, the attempt had better be omitted, for an excess of zeal in this direction exposes the physician to the commission of some of the sweet-scented and delicately tinted errors which characterize our neighbors of sugar-pellet notoriety.

Now, we are impressed with the belief that the profession is threatened by a danger emanating from apparently a friendly source. We refer to the possibility of deception in regard to the quality of many of the "elegant" pharmaceutical preparations which are being so assiduously introduced to the notice of the medical profession, and to the notice of the general public also, by ambitious manufacturers.

It is not our purpose to impute fraud to these enterprising gentlemen, but, judging from the testimony which they themselves supply, it must be admitted if all are believed, few only can be trusted, and when self-interest is the prime motive, the public good must of necessity be only of secondary consideration, vehement protestations to the contrary notwithstanding. But leaving out of view the question of fraud, which we seriously cannot believe amounts to much, can it always be known that these physic mills furnish precisely what is claimed, and what in good faith the proprietors think they are furnishing?

We ask the question, not with the intention of returning a categorical answer at present, but for the purpose of suggesting merely a probable danger and a possible cause of a certain amount of the prevailing therapeutic scepticism of the day.

So much then for those compounds which carry their formulæ upon their face, and now what shall we say for another class of products? The connecting link between the "elegant" and the proprietary preparations, or, in other words, the would-be professional nostrum. Those compounds, for instance, provided for convenience, with a sort of *E pluribus unum* name—a two-handled cognomen, as it were—with one side labeled for "physicians use only," and the other, to be accommodating and to save trouble, within easy reach of the dear people!

We cannot regard the resort to these very questionable proceedings otherwise than an encouragement to quackery, or otherwise than a modified nostrum traffic, differing in no material point from the ordinary trade in the grossest proprietary compounds, and differing at all only in the method of their attempted introduction and the professions which accompany them. The principle which underlies the business is the same; the evil is as great or greater, for the use of these ready made prescriptions—these secret remedies—is liable to serious abuse in the hands of the people, and urged upon the attention of the public, especially accompanied by the expressed or implied approval of respectable practitioners, tend directly to promote the use of straight out nostrums, to injure the medical profession, to bring disrepute upon its members and discredit upon its practices. Therefore, we hold that medical men should set their faces against these harmful innovations and, by rejecting this class of preparations, refuse to lend aid or encouragement, immediate or remote, to a scheme at variance with their own interests and with the best interests of society. A scheme devised and executed solely in the interest and for the behoof the producer.

The medical profession can well afford to be something higher and something better than mere nostrum vendors, and we trust that the evil indicated may be promptly nipped in the bud, even at the expense of inflicting bitter disappointment and pecuniary loss upon a certain set of commercial gentlemen whose impatience to reap golden harvests and to grow rapidly rich, rather than a purely disinterested and philanthropic desire to extend and improve our materia medica, inducts them into profitable but positively pernicious practices.

A word to the wise is sufficient.

